# Peritoneal Tuberculosis Presenting as Massive Ascites in Pregnancy: A Case Report

**DOI:** 10.1002/ccr3.73249

**Published:** 2026-07-29

**Authors:** S. Gyamtsho, S. Dechen, S. Choden

**Affiliations:** ^1^ Department of Obstetrics and Gynecology Jigme Dorji Wangchuck National Referral Hospital Thimphu Bhutan; ^2^ Faculty of Postgraduate Medicine Khesar Gyalpo University of Medical Sciences of Bhutan Thimphu Bhutan; ^3^ Department of Pathology and Laboratory Medicine Jigme Dorji Wangchuck National Referral Hospital Thimphu Bhutan

**Keywords:** ascites, diagnosis, peritoneal tuberculosis, pregnancy

## Abstract

Abdominal tuberculosis, especially peritoneal tuberculosis, is uncommon in pregnancy. The diagnosis during pregnancy is a challenge due to many signs and symptoms being nonspecific and mimicking the symptoms of pregnancy. The presence of ascites, with anorexia and weight loss in pregnancy from endemic regions, should be addressed with suspicion of abdominal tuberculosis.

## 1 Introduction

Tuberculosis (TB) caused by Bacillus *Mycobacterium tuberculosis* leads to pulmonary as well as extra‐pulmonary TB which includes pleura, abdomen, spine, urogenital tract, and central nervous system known as extra‐pulmonary TB [1]. Abdominal tuberculosis, especially peritoneal tuberculosis, is uncommon in pregnancy [2].

Tuberculosis is a leading cause of global morbidity and mortality, with an estimated 10.6 million new cases, and 16% had extra‐pulmonary tuberculosis; abdominal tuberculosis constitutes about 10% of extra‐pulmonary tuberculosis [3, 4]. More than 200,000 pregnant women become sick with TB each year, and it is one of the non‐obstetric causes of maternal mortality, the majority in resource‐limited countries [5]. TB in pregnancy is associated with increased chances of abortion, preterm labor, fetal prematurity, low birth weight babies, preeclampsia, and postpartum hemorrhage [6]. Here, the authors present a case of peritoneal TB in pregnancy presenting with ascites, abdominal pain, weight loss, and who had a premature delivery.

## 2 Case History

A 30 years old multiparous, at 36 weeks of gestation with a previous history of caesarean section, presented to Jigme Dorji National Referral Hospital with anorexia, poor weight gain, and abdominal distension causing discomfort.

She was referred to this tertiary Hospital as a case of massive ascites in pregnancy causing abdominal discomfort. She had progressive distension of abdomen during the later part of pregnancy, poor weight gain and preterm labor. She denied having cough but had night sweating. None of the family members had suffered from tuberculosis and had been vaccinated with Bacillus Calmette‐Guerin (BCG) vaccine as a child.

**FIGURE 1:**
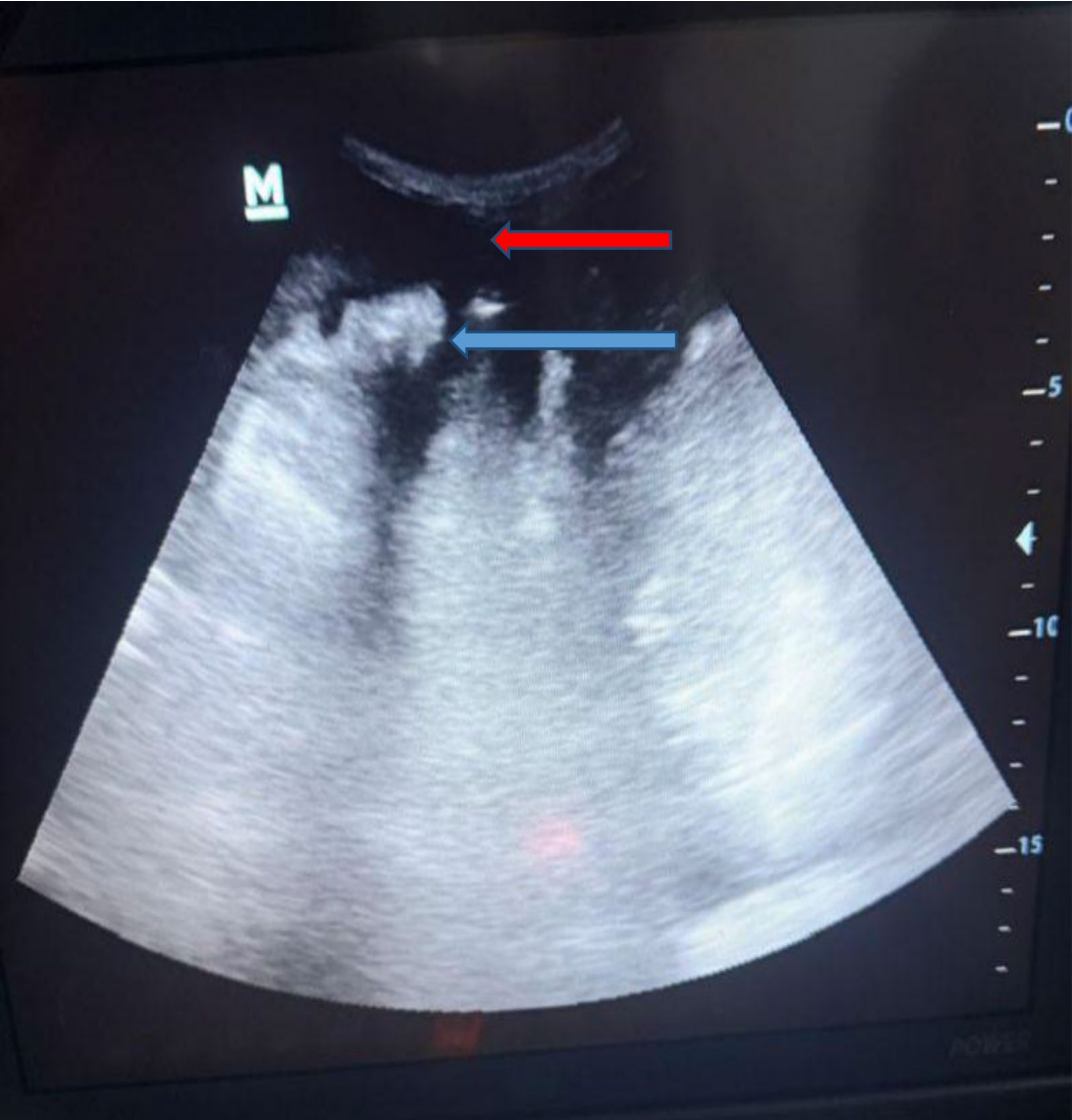
Presence of ascites (red arrow) and thickening of omentum (blue arrow) and bowel wall.

On clinical examination, she had discomfort on lying down due to abdominal distension: Pulse rate 76/min, blood pressure 120/70 mmHg, no edema, no pallor, or jaundice. Abdomen was grossly distended and difficult to palpate the fetal parts. An ultrasound scan done, revealed the presence of ascites, with thickening of omentum and bowel walls (Figure 1), and presence of a live preterm fetus in the uterine cavity.

## 3 Investigation, Differential Diagnosis, and Management

The blood investigation showed a total leucocyte count of 8.99 × 10^3^/μL, neutrophil 77.8%, lymphocyte 14.44%, and hemoglobin of 10.70 g/d L. Liver and renal function tests were within normal range. Sputum was negative for acid‐fast bacilli. Chest X‐ray done showed no abnormality except for a few calcifications.

**FIGURE 2:**
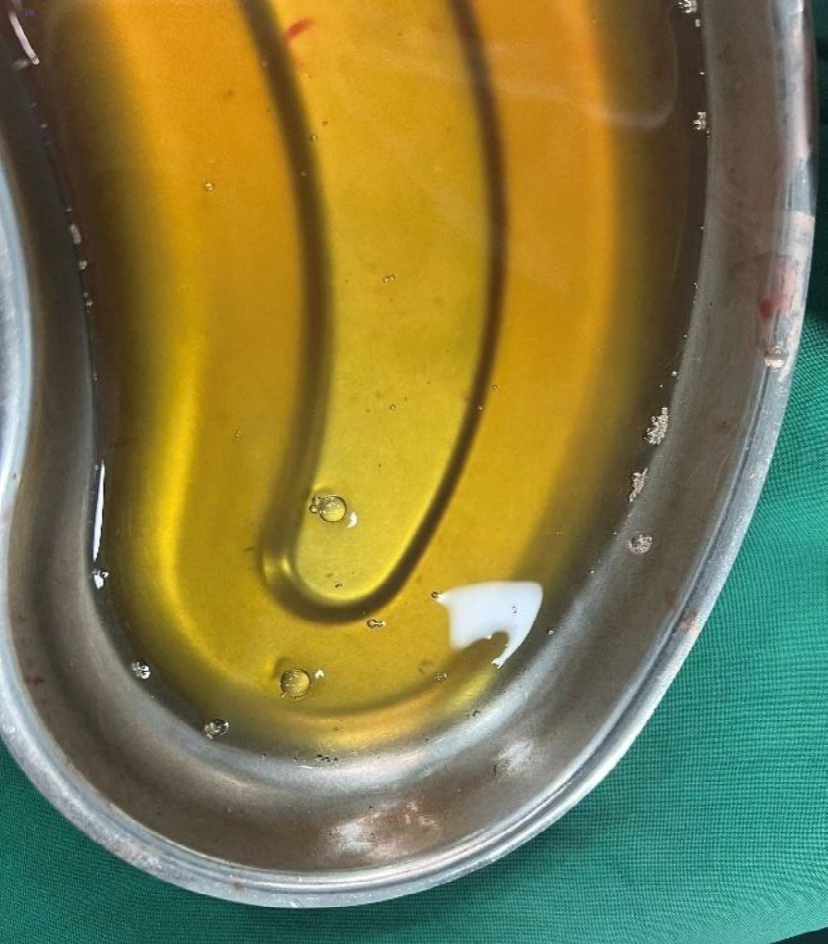
Straw colored ascites fluid drained out from the abdominal cavity (the white colored in the straw colored fluid is an artifact).

She underwent emergency caesarean section at 36 weeks, due to preterm labor caused by abdominal distension. Intraoperatively, about 1.5–2 L of straw colored ascitic fluid (Figure 2) was drained out before the delivery of the baby.

**FIGURE 3:**
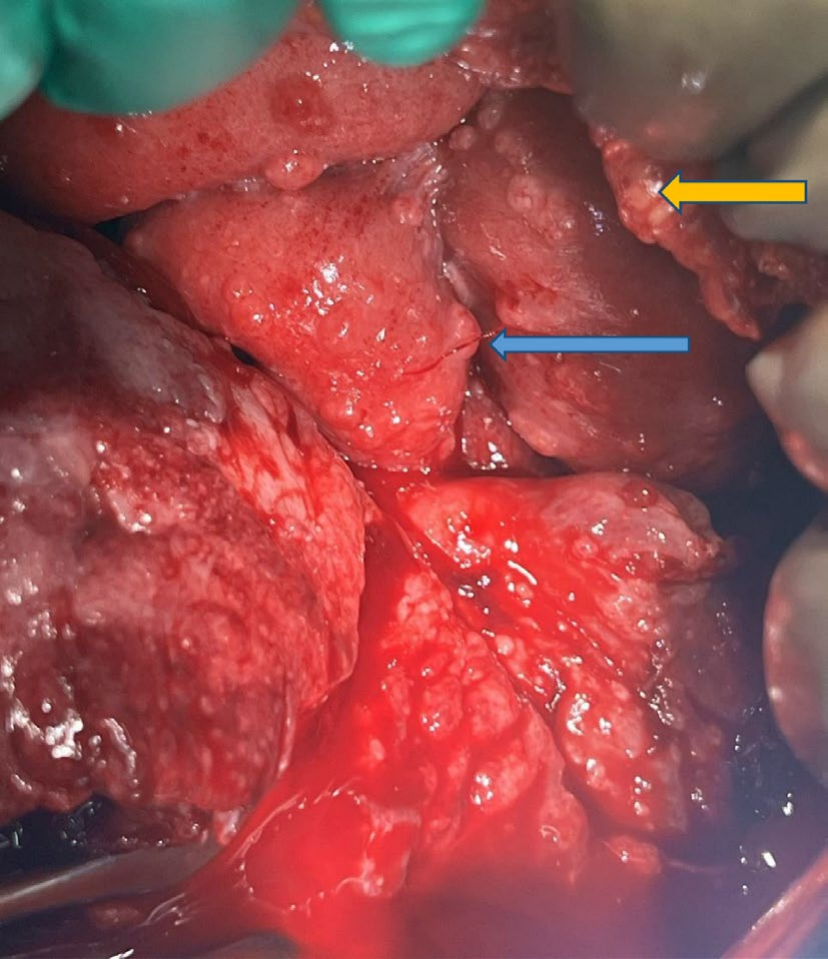
Nodular deposits on the bowel surfaces (blue arrow) and parietal peritoneum of abdominal wall (yellow arrow).

There were presences of many yellowish white nodular deposits on the surface of the uterus, bowels, omentum and parietal peritoneal surfaces which were suspicious of tuberculosis (Figure 3).

A male fetal growth restricted baby weighing approximately 2 kg was delivered.

## 4 Conclusion and Results

A provisional diagnosis of peritoneal tuberculosis in pregnancy was made based on the clinical examination of the nodular deposits.

**FIGURE 4:**
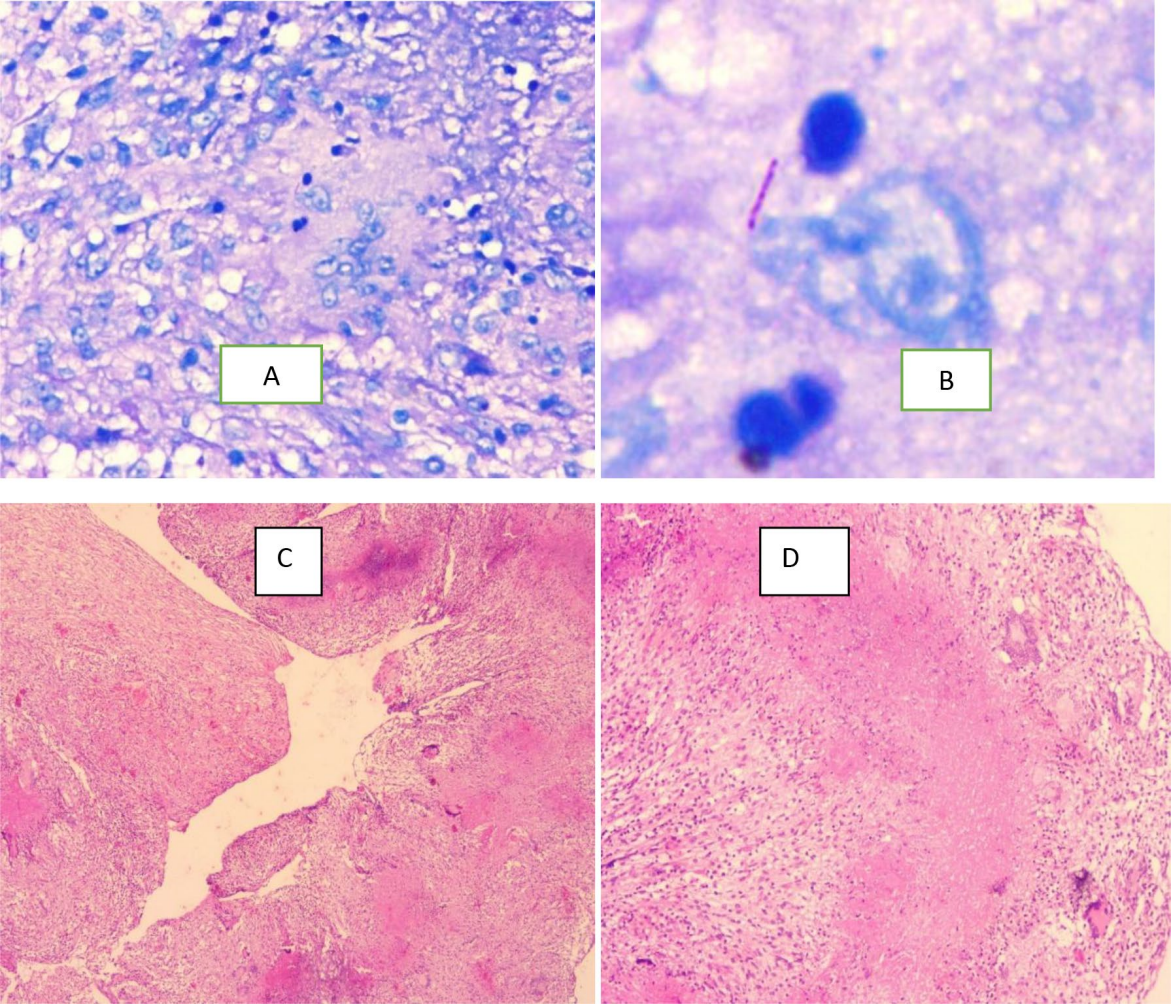
Microscopic image of abdominal wall lesion stained with Ziehl–Neelsen stain highlighting one acid‐fast bacilli (A, B), and abdominal wall lesion stained with hematoxylin and eosin displaying foci of caseating necrosis surrounded by epithelioid histocytes with occasional multinucleated giant cell of histiocytes (Langhan‐type) (C, D).

Cytology examination of the ascitic fluid revealed scanty cellularity containing macrophages, few mesothelial cells, and few lymphocytes suggestive of chronic inflammation. No malignant cells were seen. GenXpert report from the deposits was positive for *Mycobacterium tuberculosis* . The Ziehl–Neelsen staining showed scanty presence of acid fast bacilli (Figure 4A,B), and histopathology examination of the biopsy revealed the presence of caseating necrosis and presence of Langhan‐type giant cells (Figure 4C,D).

All these investigations confirmed it as a case of extra‐pulmonary (abdominal) tuberculosis. A short course regime (6 months regime) consisting of 2 months of combination of isoniazid (INH), rifampicin (RIF), ethambutol (EMB), and pyrazinamide (PZA) daily for 2 months followed by INH, RIF, and EMB for 4 months was started.

She was discharged on post‐operative day 7 after removal of abdominal skin sutures. She was followed up in the post‐natal clinic. She was responding to anti‐tuberculosis treatment without any complications or side effects. The baby was not put on INH prophylaxis as the mother's sputum was negative for TB. She was advised non‐hormonal contraception at 6 weeks.

At the time of writing of this case report, the patient was on anti‐tuberculosis treatment and was in stable condition and had good compliance with the intake of medications.

## 5 Discussion

Globally more than 220,000 pregnant women are diagnosed with active tuberculosis, of which 10 to 20% are extra pulmonary tuberculosis, and the greatest burden is in African and Southeast‐Asian regions [7]. Tuberculosis in pregnancy poses a substantial risk of morbidity and mortality to both the pregnant women and the fetus if not diagnosed and treated in time. Abdominal tuberculosis, especially peritoneal tuberculosis, is uncommon in pregnancy [2].

Pregnant women are more susceptible to new infections and reactivation of tuberculosis due to pregnancy which suppresses the T‐helper cells and its pro‐inflammatory response [8]. The current theory for peritoneal tuberculosis development is due to direct spread into the peritoneum from the fallopian tubes or from the hematogenous or lymphatic route or reactivation of the latent tuberculosis. Peritoneal tuberculosis is further classified into dry and wet types depending on the presence or absence of ascites. The wet type is the most common and occurs in 90% of the cases of peritoneal tuberculosis [9]. Our case does not have a pulmonary focus and could be due to reactivation of latent tuberculosis and is a wet type of peritoneal tuberculosis due to the presence of massive ascites. Peritoneal tuberculosis with pregnancy is very rare as this type of tuberculosis is known to cause infertility [10].

The symptoms of peritoneal tuberculosis are abdominal pain, distension, fever, weight loss, diarrhea/constipation in most of the cases [10]. Diagnosis of peritoneal tuberculosis is often delayed in pregnancy due to nonspecific symptoms such as nausea, loss of appetite, weakness, weight loss (or poor weight gain) and abdominal distension which is also seen in pregnancy [6, 7]. Peritoneal tuberculosis should be suspected when ascitic fluid is detected during pregnancy [11]. In our case, the patient presented with abdominal distension and discomfort, but she didn't have fever or diarrhea or constipation. Because of the nonspecific nature of symptoms associated with abdominal tuberculosis, there was a delayed diagnosis in pregnancy and diagnosed intraoperatively. The majority of the patients of extra‐pulmonary tuberculosis do not have a pulmonary focus or contact which is similar in our case.

The diagnosis of abdominal tuberculosis remains one of the most challenging tasks in clinical practice. The presence of nodule deposits in the peritoneal surfaces and bowels does lead to the differential diagnosis of malignancy or carcinomatosis [10]. Collection of peritoneal fluid and sending for investigation for cytology and for presence of acid‐fast bacilli (AFB), including histology examination of the biopsy from the nodular deposits, where appropriate and service available. WHO recommends GenXpert *Mycobacterium tuberculosis* (MTB) assay for diagnosing tuberculosis. It can provide results within 2 h. GenXpert test has high specificity but limited sensitivity for detecting extra‐pulmonary [12]. Histology pathology examination of the tissue biopsy will show the presence of caseous necrosis and presence of Langhans giant cell, which is suggestive of tuberculosis [11]. The Ziehl–Neelsen staining done from the tissue can show the presence of AFB suggestive of *Mycobacterium tuberculosis* [12]. In our case from the multiple biopsies taken from the peritoneal nodules, GenXpert test done showed the presence of MTB and Ziehl–Neelsen staining showed very few AFB suggestive of tuberculosis. The histopathology examination showed presence of caseous necrosis and giant cells highly suggestive of peritoneal tuberculosis. No malignant cells were detected on histopathology ruling out any presence of malignancy.

Adverse maternal and neonatal outcomes are increased with inadequate treatment, advanced disease, and late diagnosis of tuberculosis in pregnancy compared with earlier diagnosis [7]. Abdominal tuberculosis is known to cause the increased risk of miscarriage, fetal growth restriction, prematurity, and risk of preeclampsia [5, 11]. Infants born to mothers with tuberculosis have a higher incidence of congenital defects [13]. In our case, a late premature baby weighing 2 kg at birth was delivered, with features of fetal growth restriction, but no congenital defects. Studies have shown that the risk of death for mothers with tuberculosis increases, and these mothers are more likely to have complications such as gestational hypertension, rupture of pulmonary bullae, postpartum hemorrhage, sepsis, anemia, placental chorioamnionitis, and gestational diabetes [12, 13]. There were no adverse outcomes both to the mother and the neonate after birth in our case and both were discharged from our hospital after 7 days post‐delivery.

## 6 Conclusion

Diagnosis of abdominal tuberculosis (peritoneal tuberculosis) is a challenge due to some of the signs and symptoms overlapping with non‐specific features of pregnancy. The presence of ascites, with anorexia and weight loss in pregnancy from an endemic region, should be addressed with suspicion of abdominal tuberculosis. Accurate diagnosis requires isolation of *Mycobacterium tuberculosis* from peritoneal biopsy and histopathologic examination of the biopsy from peritoneal nodules. Diagnosis and proper management of extra pulmonary TB in pregnancy will prevent adverse obstetrics and neonatal morbidities and mortality.

## Author Contributions

**S. Gyamtsho:** conceptualization, methodology, writing – original draft, writing – review and editing. **S. Dechen:** writing – review and editing. **S. Choden:** writing – review and editing.

## Funding

The authors have nothing to report.

## Consent

Written informed consent was obtained from the individual for the publication of the case and the images included in this article.

## Conflicts of Interest

The authors declare no conflicts of interest.

## Data Availability

The authors have nothing to report.
